# Our Ensign

**Published:** 1914-09

**Authors:** 


### Our Ensign.

The ensign of the TEXAS MEDICAL JOURNAL is Helpfulness, Progress and Originality. These three will be stressed. It is earnestly desired that the JOURNAL may first of all be helpful to both readers and advertisers. The most advanced thought of the day will be given in every issue of the JOURNAL. This can be done by reviewing all progressive journals and magazines and by securing papers from advanced thinkers. The average physician has little time to read the current literature of the day or to read half dozen medical journals, so it is intended to cull from the pages of other magazines that which you should know and make a digest of it so that you may be able to form your own conclusions of the matter regarding the merits of the question under discussion.

The things that are new in medicine today are old tomorrow, so great are the strides being made in materia medica.

If you will read over carefully the list of contributing editors, you will agree that men have been selected with strength of character and of mind. And remember they are really going to write; they have promised to write at least two articles.

If there is any particular subject that you would have any one of them discuss, if you will write the "Red Back" and request it an earnest effort will be made to get the doctor to write on the subject that you may choose for him. Now, is not that fair and a most unusual opportunity? You choose the subject in which you are most interested and the man who you wish to have write upon that subject.

The JOURNAL is always open to the subscribers to ask questions or to discuss any question or to comment upon anything appearing in the columns of the same. Of course, all discussion will be kind and courteous, and while you have a perfect right to disagree with the writer you can of course state your disagreement in kindly and gentlemanly terms. You are cordially invited to ask questions of any writer or to discuss any article written. Then there is one other thing that you must not forget, if you would make the JOURNAL really helpful, and that is to praise those who do well for you. If you write a good, strong paper, you appreciate a card from some fellow physician telling you how much he enjoyed the paper, don't you? Well, remember that every writer appreciates deserved praise.

The reading matter and the advertisements make up the JOUR-

nal, and it is striving just as hard to give you the best in advertising as in the reading matter. Please do not get the idea that the advertising is carried purely for the money there is in it. You may not know that there are firms advertising medicines that could not buy one inch of space in this JOURNAL not for all the money they possess. The JOURNAL, as we have said before, is to be, first of all, helpful, and, if it is to be helpful to you, there shall no fraudulent advertisements appear in its pages. The things you need and use are not advertised in the newspapers and current magazines; therefore, the JOURNAL becomes an up-to-date directory to keep you informed of the progress that is being made in the great chemical laboratories of the world. In soliciting advertisements it is always borne in mind that some reader of the JOURNAL may be wanting and needing this very thing, and that the advertisement will prove helpful both to advertiser and to reader.

You cannot afford to miss reading the advertisements any more than you can afford to miss the strongest articles, for the adver-vertisers are wide awake, and each month there is added something new in the drug world and the clean progressive advertiser knows that the average doctor is keeping up and he tries to help you by giving you the best that can be had on the market.

The doctor who fails to read the advertisements in a clean, reliable journal is standing in his own light, and it will only be a short time until the profession and the community in which he lives will brand him an old fogy, and he deserves it. For, at a great expense, the advertisers are preparing new and better remedies and are refusing to advertise these remedies in the newspapers and current magazines. How then is the public to be benefited by these remedies if the physicians do not keep informed?

It is just as essential to help advertisers as to help readers, and to this end all advertisements must be honest and beneficial, and of a character to interest the readers of the TEXAS MEDICAL JOURNAL and the public its readers serve.

An advertiser is known by the company he keeps, and the "Red Back" proposes to protect the good standing of its advertisers to the extent of its ability and calls upon all its readers to help do this.

It is believed by this JOURNAL that in giving advertisers an enlightened and progressive patronage that they are giving the best possible service and that in turn the advertisers will give the appreciative physician the best in their power.

Advertisers are human, just like the rest of mankind, and while they are more accustomed to hear from those whom they serve, only when they have a complaint to make, they would appreciate a kind word now and then from those whom they serve so faithfully. Suppose as a family we try words of commendation rather than censure, and see if the world does not look brighter for us.

